# Experimental procedures for flow cytometry of wild-type mouse brain: a systematic review

**DOI:** 10.3389/fimmu.2023.1281705

**Published:** 2023-10-30

**Authors:** Robert C. Sharp, Dylan T. Guenther, Matthew J. Farrer

**Affiliations:** Department of Neurology, McKnight Brain Institute, University of Florida, Gainesville, FL, United States

**Keywords:** neuroimmune, flow cytometry, mouse studies, immunity, systematic review, inflammation, neurological disorders, methodology

## Abstract

**Objective:**

The aim of this study was to systematically review the neuroimmunology literature to determine the average immune cell counts reported by flow cytometry in wild-type (WT) homogenized mouse brains.

**Background:**

Mouse models of gene dysfunction are widely used to study age-associated neurodegenerative disorders, including Alzheimer’s disease and Parkinson’s disease. The importance of the neuroimmune system in these multifactorial disorders has become increasingly evident, and methods to quantify resident and infiltrating immune cells in the brain, including flow cytometry, are necessary. However, there appears to be no consensus on the best approach to perform flow cytometry or quantify/report immune cell counts. The development of more standardized methods would accelerate neuroimmune discovery and validation by meta-analysis.

**Methods:**

There has not yet been a systematic review of ‘neuroimmunology’ by ‘flow cytometry’ via examination of the PROSPERO registry. A protocol for a systematic review was subsequently based on the Preferred Reporting Items for Systematic Reviews and Meta-Analyses (PRISMA) using the Studies, Data, Methods, and Outcomes (SDMO) criteria. Literature searches were conducted in the Google Scholar and PubMed databases. From that search, 900 candidate studies were identified, and 437 studies were assessed for eligibility based on formal exclusion criteria.

**Results:**

Out of the 437 studies reviewed, 58 were eligible for inclusion and comparative analysis. Each study assessed immune cell subsets within homogenized mouse brains and used flow cytometry. Nonetheless, there was considerable variability in the methods, data analysis, reporting, and results. Descriptive statistics have been presented on the study designs and results, including medians with interquartile ranges (IQRs) and overall means with standard deviations (SD) for specific immune cell counts and their relative proportions, within and between studies. A total of 58 studies reported the most abundant immune cells within the brains were TMEM119^+^ microglia, bulk CD4^+^ T cells, and bulk CD8^+^ T cells.

**Conclusion:**

Experiments to conduct and report flow cytometry data, derived from WT homogenized mouse brains, would benefit from a more standardized approach. While within-study comparisons are valid, the variability in methods of counting of immune cell populations is too broad for meta-analysis. The inclusion of a minimal protocol with more detailed methods, controls, and standards could enable this nascent field to compare results across studies.

## 1 Introduction

The mouse has been used to model neurological disorders for many decades, whether employing lesion models (i.e., inducing a stroke in a mouse specimen) or transgenic or more physiologic gene knock-out or mutant knock-in approaches (1–3). Some illustrations of mouse modeling for neurologic and neurodegenerative disorders include work in Alzheimer’s disease (AD), Parkinson’s disease (PD), Huntington’s disease (HD), multiple sclerosis (MS), psychological/intellectual disabilities (i.e., depression or Down’s syndrome), traumatic brain injuries (TBI), and prion diseases (1–3). Most of these disorders are multifactorial, with genetic and environmental components, for which the immune system may provide some integration. Consequently, there has been growing interest in neuroimmunology that is not just focused on resident immune cells within the brain but also on infiltrating peripheral immune cells (4–7). Recent studies of the gut microbiota have highlighted nervous and immune communication between the gut and central nervous system (CNS) (8–10). With this resurgence, researchers have employed traditional (i.e., immunofluorescence/histology slide staining and Western blotting) and contemporary methods (i.e., single-cell genomics and single-cell sorting/staining via flow cytometry) to better characterize specific immune cell subsets within the body and brain.

Specifically, brain resident immune cells, including microglia and astrocytes, have been comprehensively examined in many mouse models of neurologic disorders (4–10). However, the characterization of other immune cell subsets in peripheral blood, the CNS, and within the brain (both residential and infiltrated) has yet to be fully described (4–10). These immune cell subsets include, but are not limited to, natural killer cells (NK cells), T cells, and B cells (4–10). However, validation and comparison through meta-analysis of immunophenotypes of these mouse models might be enabled if researchers utilize more standardized methods and reporting.

Technologic developments for single-cell isolation and analysis, including single-cell RNAseq, mass cytometry (CYTOF), fluorescence-activated cell sorting (FACS), and multi-color flow cytometry-derived immunophenotyping, have illuminated a wide variety of scientific fields (11, 12). Of these techniques, FACS and flow cytometry immunophenotyping are most frequently used. Reasons include the ease of use in setting up a flow cytometer or sorter for a variety of applications, the sensitivity of the assay, the specificity of the antibodies used, the potential of those antibodies to be used for both flow cytometry and Western blotting for the same target, and the ability to produce qualitative and quantitative data (12, 13).

Nevertheless, flow cytometry has its pitfalls as analytic interpretation of the data depends on the user’s preference for gating and target choices (13). Experimentally, flow cytometry is also dependent on the fluorophores and cytometers used, and variation between these instruments may result in false positives and negatives. Nevertheless, such issues can be circumvented by providing multiple controls, such as unstained, isotype antibody-stained, and fluorescence-minus-one (FMO) controls, more rigorously described methods, and standardization of flow cytometry protocols.

FACs and flow cytometry immunophenotyping have been insightful and widely used in basic immunology, and other scientific fields, including neurology, have begun to adopt these methods (14–16). However, now that their utility has been demonstrated, the use of flow cytometry for detecting cell types within the brain and CNS warrants more standardized protocols and reporting. Although mouse immune profiles within the brain have been identified by high-dimensional single-cell mapping using techniques such as mass cytometry (CYTOF) (17), at the time of writing and despite the numerous publications, the expected cell counts and proportions of each immune subset within the brain of a wild-type (WT) mouse have not yet been clearly defined by standard FACS and flow cytometry. Consequently, we have performed a systematic review focused on neuroimmunology and the use of flow cytometry to detect immune cells derived from WT homogenized mouse brains. In our results, we summarize the number of immune cells overall and estimate the immune subsets that can be detected via flow cytometry immunophenotyping. Our findings demonstrate a critical need for more standardized methods and reporting and lead to best-practice recommendations for future publications.

## 2 Methods

### 2.1 Study design

The study design was informed by prior literature (18–20) and based on the Preferred Reporting Items for Systematic Reviews and Meta-Analyses (PRISMA) criteria (21) and the Cochrane Handbook of reporting methodology reviews, employing the SDMO (Types of Studies, Types of Data, Types of Methods, and Types of Outcome Measures) criteria (22, 23). Bias assessment for each individual study selected for systematic review inclusion was also conducted using the Systematic Review Centre for Laboratory animal Experimentation (SYRCLE) (24) methodology, and subsequent reporting used the *robvis* R package and Shiny web app (25).

### 2.2 Search strategy

For this systematic review, the Google Scholar and PubMed databases were used to identify all studies published between January 2013 and July 2023. The search protocol and study design were also assessed within the National Institute of Health (NIH) PROSPERO registry database, which confirmed that a review of this topic has not been previously conducted. For database searches, the following combination of keywords was used to identify eligible studies: “flow cytometry” AND “immune subset name examined in study” AND “mouse brain.” For example, a keyword search containing “flow cytometry CD4 T cells mouse brain” was used. “Immune subset name examined in study” is defined as one of the following immune cell subsets: “CD4 T cells,” “CD8 T cells,” “double negative DN T cells,” “regulatory T cells TREG,” “follicular helper T cells TFH,” “T helper 1 T cells TH1,” “T helper 2 T cells TH2,” “T helper 17 T cells TH17,” “naïve T cells,” “naïve CD4 T cells,” “naïve CD8 T cells,” “naïve-like T cells,” “central memory T cells TCM,” “central memory CD4 T cells TCM,” “central memory CD8 T cells TCM,” “effector memory T cells TEM,” “effector memory CD4 T cells TEM,” “ effector memory CD8 T cells TEM,” “effector memory T cells re-expressing CD45RA TEMRA,” “effector memory CD4 T cells re-expressing CD45RA TEMRA,” “effector memory CD8 T cells re-expressing CD45RA TEMRA,” “TEMRA-like T cells,” “natural killer cells NK cells,” “dendritic cells DC,” “B cells,” “monocytes,” “macrophages,” “M1 macrophages,” “M2 macrophages,” “TMEM119 microglia,” or “neutrophils.”.

### 2.3 Selection and exclusion criteria

Studies were selected from the search results employing the following inclusion criteria: 1) any study performed between 2013 and 2023; 2) any study that contained flow cytometry data identifying immune cell subsets and counts in mouse brains; 3) any study that reported total cell numbers or total live cell percentages for one or more immune cell subsets; and 4) any study that had WT mice or a non-treated control (when reporting on transgenic mouse models). Studies were excluded from search results based on the following criteria: 1) studies performed in 2012 and prior; 2) any study not focused on flow cytometry of homogenized mouse brains; 3) rat studies; 4) human studies; and 5) studies not reporting controls or the background mouse strain.

### 2.4 Data extraction

For each study, information on the mouse strain used, age, and sex was recorded. In addition, methodological information on mouse perfusion, brain extraction, and homogenization was recorded. Flow cytometry methods were recorded when the following was reported: 1) cytometer make and model; 2) software for data collection/analysis; 3) full gating strategy; 4) total cells collected per sample; 5) total live cell counts; 6) total immune cell subset percentages calculated directly from live cell counts; and 7) methods for determining cell counts and/or mean fluorescence intensity (MFI) readings.

Studies were subsequently examined for immune cell subset counts from WT/control mouse brains directly from the main text, **Supplementary Materials**, and/or extrapolated from the figures and graphs presented. In this systematic review, “raw” total immune cell subset counts were reported for each immune cell subset as a median with an interquartile range (IQR; defined as 75^th^ percentile upper quartile [Q3] – 25^th^ percentile lower quartile [Q1]) and as the combined mean with standard deviation (SD) of multiple studies (n). The “raw” total overall cell count collected per sample by flow cytometry was also reported from these studies.

### 2.5 Data analysis

After the median with IQR and combined means with SD from the “raw” total immune cell subset counts and from the “raw” total overall cell count collected per sample were recorded from each study, we standardized an approach to estimate how many immune cells of each subset would be counted if the total homogenized mouse brain cells collected by flow cytometry equaled a total of 1 x 10^5^ collected cells. The value of cells required per sample for an accurate flow cytometry reading is stated to be between 1 x 10^4^ total cells (minimum) and 1 x 10^6^ total cells (maximum); hence, we used the median (26).

The standardized total cell counts for each immune cell subset reported in this systematic review were calculated using the following equation:


$$\frac{a}{b}=\frac{X}{1\times 10^{5}Total\;Cells\;Collected}$$

Where “a” is the [Raw Total Immune Cell Subset Count], “b” is the [Raw Total Cell Count Collected] per sample, and “X” is the [Standardized Total Immune Cell Subset Count]. Rearranging to solve for X gives the following equation:


$$\frac{\left(a)(1\times 10^{5}Total\;Cells\;Counted\right)}{b}=X$$

Once all total immune cell subset counts were standardized, we determined the theoretical standardized percentages of each immune cell subset within the entire WT mouse brain and reported those results as means with SD.

## 3 Results

### 3.1 Literature search and study selection

**Figure 1:**
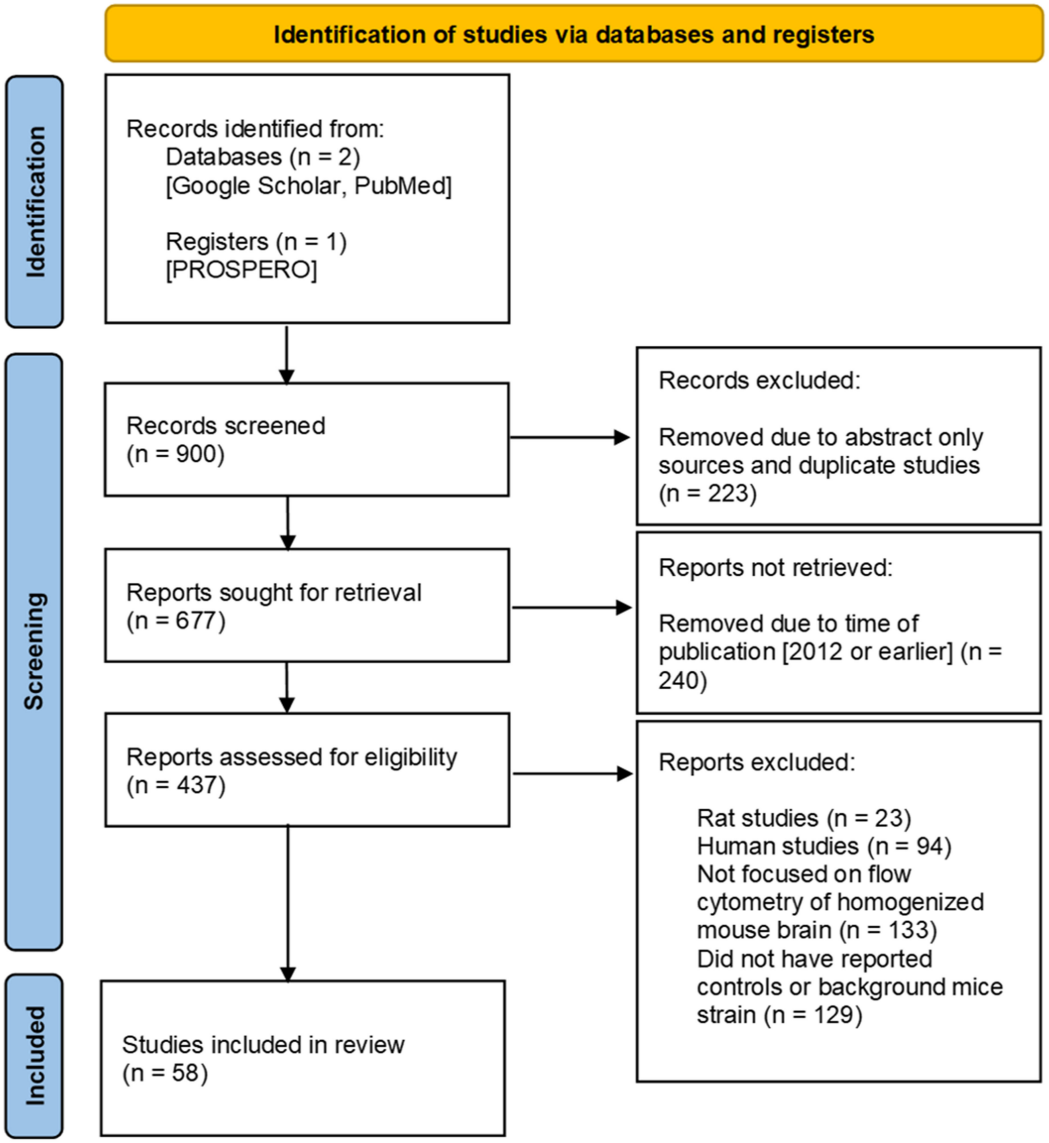
Prisma flow diagram. Guidelines provided by PRISMA (21). Work flow diagram was created by template provided by Page MJ, McKenzie JE, Bossuyt PM, Boutron I, Hoffmann TC, Mulrow CD, et al. The PRISMA 2020 statement: an updated guideline for reporting systematic reviews. BMJ 2021;372:n71. doi: 10.1136/bmj.n71.

A PRISMA-based flow diagram illustrates our screening methodology for study identification (**Figure 1**) (21). The two databases (Google Scholar and PubMed) were searched using keywords, as defined in the Methods, and 900 articles were highlighted for systematic review. Of those 900 reports, 223 were removed as they supplied only an abstract or were duplicated between databases, and 677 studies remained. Of these, an additional 240 studies were removed as they were published in 2012 or prior. This cut-off is arbitrary, but it was used to identify more contemporary flow cytometry immunophenotyping publications and resulted in 437 eligible studies. Further inclusion and exclusion criteria removed 133 studies in which flow cytometry of homogenized mouse brains was not a main focus, 129 studies with insufficient information and/or results from controls or in which the background mice strain was not specified, 94 human studies, and 23 rat studies. Consequently, 58 studies that passed our inclusion and exclusion criteria were incorporated into this systematic review (27–84).

### 3.2 Reporting of mouse information and perfusion/tissue processing is inconsistent between studies

**Figure 2:**
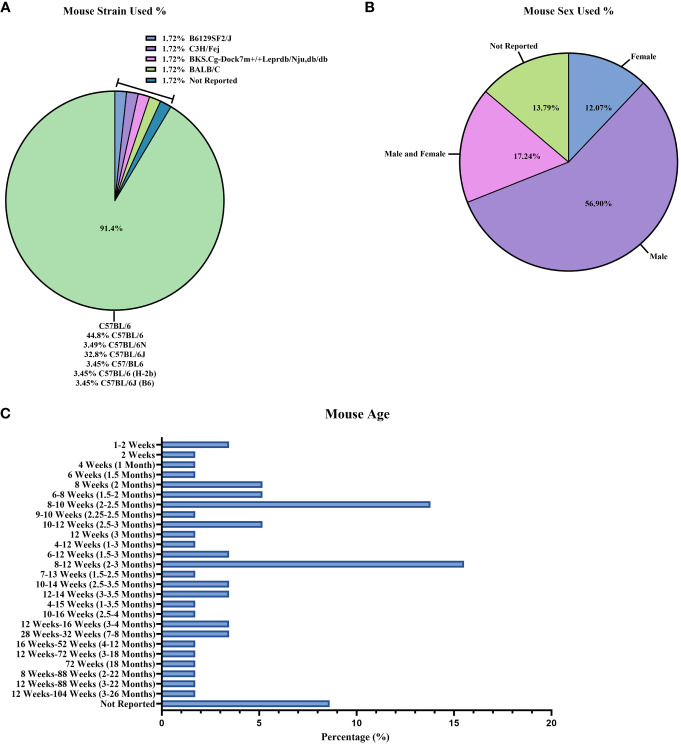
Mouse information reported between studies. Reported baseline mouse information described as percentages out of the 58 studies: **(A)** mouse strains; **(B)** mouse sex; and **(C)** mouse age.

Of the 58 studies selected, we reviewed the basic characteristics of the mice strains used (**Figure 2A**; **Supplementary Table 1**). The inbred congenic C57BL/6 mouse line was used the most (26/58 studies [44.8%]) as a control and as the background for genetically modified mice (27, 28, 30, 31, 36, 41–47, 49, 51, 54, 58, 66–69, 72, 74–76, 79, 82). However, other C57BL/6 mice sub-strains were used throughout the 58 studies, which include C57BL/6J (19/58 studies [32.8%]) (29, 32, 33, 39, 52, 56, 60, 62–65, 70, 71, 73, 78, 80, 81, 83, 84); C57/BL6 (2/58 studies [3.44%]) (34, 40); C57BL/6 (H-2b) (2/58 studies [3.44%]) (35, 53); and C57BL/6J (B6) (2/58 studies [3.44%]) (38, 61). The majority of studies that reported mouse sex (**Figure 2B**; **Supplementary Table 1**) used only male mice (33/58 studies [56.9%]) (29, 30, 33, 34, 38, 40, 41, 44, 45, 47, 49, 50, 54, 58–63, 65, 67–71, 74, 77–80, 82–84), although 10/58 studies [17.2%] reported mixed results of male and female animals together (27, 31, 32, 36, 37, 39, 43, 51, 72, 75). The age of the mice (**Figure 2C**; **Supplementary Table 1**) within the studies varied, but the majority reported findings at 8-12 weeks (2-3 months) (9/58 studies [15.5%]) (29, 33, 56, 57, 65, 72, 75, 80, 83). However, studies characterized animals over a wide range of ages, from 1 to 2 weeks (32, 39, 81) and between 3 and 26 months (37).

**Figure 3:**
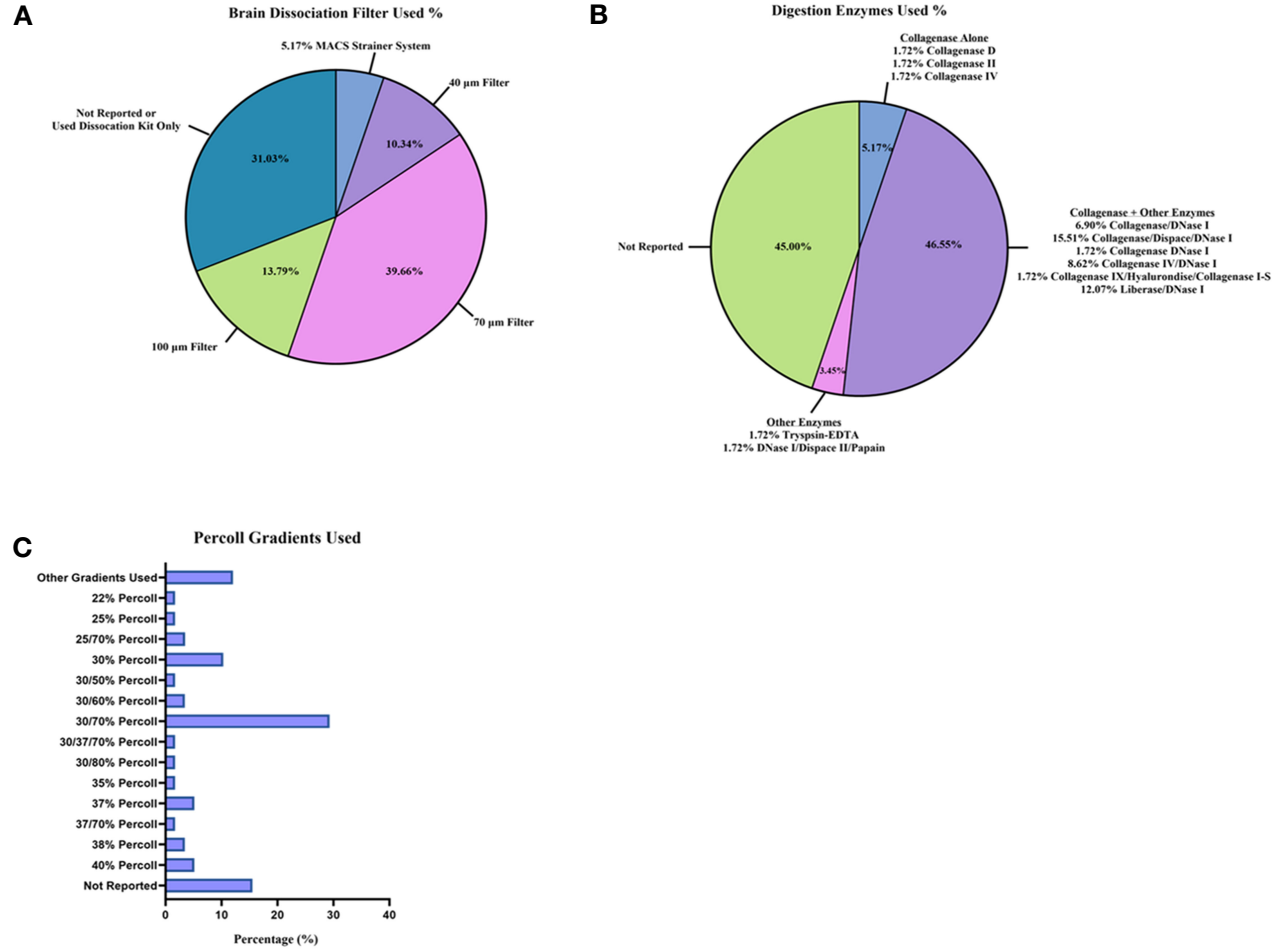
Brain tissue dissociation and cell isolation methods reported between studies. Tissue processing techniques were reported as percentages from the 58 studies: **(A)** brain dissociation filters utilized; **(B)** digestion enzymes used for brain homogenization; and **(C)** Percoll gradients used to remove myelin layer and isolate immune cells.

Perfusion (**Supplementary Figure 1**; **Supplementary Table 1**) and brain tissue processing methods (**Figures 3A–C**; **Supplementary Table 1**) also varied across the 58 studies. A majority of studies (36/58 studies [62.1%], **Supplementary Figure 1**; **Supplementary Table 1**) used cold PBS for perfusion (27, 29, 31–33, 35, 39, 43–48, 50–58, 61, 63–67, 70, 73–76, 79, 81, 84). Of the 58 studies reviewed, 6/58 [10.3%] (**Figure 3A**; **Supplementary Table 1**) used a commercial kit, such as the Neural Tissue Dissociation Kit P (Miltenyi Biotec), to isolate immune cells from mouse brain (34, 40, 41, 61, 67, 71). Nevertheless, in neurology, it remains unclear how to best process mouse brain to dissociate the tissue and leave cell types intact (85–87). Researchers may use mechanical homogenization, enzymatic homogenization, or both homogenization techniques (85–87). For the studies reviewed, 18/58 [31.0%] (**Supplementary Table 1**) used some type of mechanical homogenization (glass–Teflon homogenizer, an 18-gauge needle, etc.) before filtering through a cell strainer prior to enzyme treatment and the use of a myelin removal/immune isolation gradient (i.e., Percoll gradient) (31, 33, 37, 44, 48, 50, 53, 55–57, 59, 60, 63, 64, 66, 69, 81, 84). The enzymatic solutions used in the reports also vary widely (**Figure 3B**; **Supplementary Table 1**). Most studies used collagenase (I, II, IV, D, I-S, or Liberase) alone or combined with additional enzymes (30/58 studies [51.7%]) (27–31, 33, 46–48, 53, 54, 60, 62–64, 66–68, 70, 73–80, 82–84). The enzyme most used in combination with collagenase (or another tissue digestion enzyme) was DNase I (27/58 studies [46.6%]) (27–31, 47, 48, 51, 53, 54, 60, 62, 63, 66, 68, 70, 73–80, 82–84). After the homogenization of mouse brain, cell strainers are generally used to remove dead cells and myelin (**Figure 3A**; **Supplementary Table 1**), and a 70 μm filter was used in the majority (23/58 studies [39.7%]) of studies (15, 30, 32, 33, 38, 39, 43, 45, 46, 48, 52, 53, 56, 58, 59, 62–64, 67, 72, 74, 82, 84). To further remove myelin from mouse brain homogenate along with isolating immune cells, researchers employ a myelin removal kit or Percoll gradient solutions. Specific cell types can be isolated while the myelin layer rises to the top of the sample tube with a centrifuge, depending on the percentage of Percoll. Again, in the studies reviewed, the percentages of Percoll (**Figure 3C**; **Supplementary Table 1**) varied, with most using a 30%/70% Percoll gradient solution (17/58 studies [29.3%]) (15, 27, 29, 33, 43, 45, 53, 57, 62, 63, 71, 72, 74, 77, 79, 81, 84). Overall, the age of mice and methodology for isolating immune cell counts from the brain for flow cytometry varied greatly between the studies.

### 3.3 Flow cytometry methodology used and cell counts are inconsistently reported between studies

**Figure 4:**
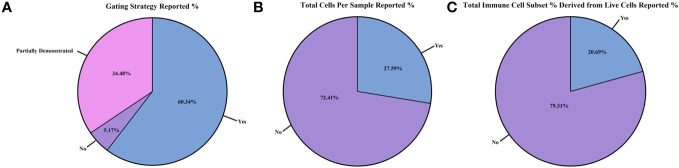
Reporting of flow cytometry results and cell counts between the studies. Differences in flow cytometry results and cell counts reported as within study percentages (n=58 studies): **(A)** gating strategy reported; **(B)** total cells per sample collected from flow cytometer/sorter reported; and **(C)** total immune cell subset counts derived from the live cell gate reported.

After considering mice strain and brain homogenization methods, we examined the flow cytometry instruments used and data reporting (**Figures 4A–C**; **Supplementary Figures 2A, B**, **3A, B**; **Supplementary Table 2**). The make and model of the flow cytometer/FACS sorter (**Supplementary Figure 2A**; **Supplementary Table 2**) and analysis software (**Supplementary Figure 2B**; **Supplementary Table 2**) used in each study also varied greatly. The flow cytometer most reported was the BD LSRII Flow Cytometer (12/58 studies [20.7%]) (30, 32, 33, 35, 36, 39, 43, 51, 60, 61, 63, 65), whereas the FACS sorter was the BD FACS Aria III (8/58 studies [13.8%]) (31, 38, 47, 49, 54, 55, 64, 83). The analysis software most generally used was a version of FlowJo (Tree Star) for flow cytometry immunophenotyping (38/58 studies [65.5%]) (27, 29–31, 33–36, 38, 41, 43–45, 47, 49–54, 56, 58, 62, 63, 65, 68–74, 76, 77, 79, 81, 83, 84) and BD FACSDiva specifically for FACS analysis (11/58 studies [19.0%]) (32, 35, 37, 39, 42, 45, 60–62, 76, 77).

After reviewing the methodology in all 58 studies, we assessed the quality of flow cytometry reporting (**Figures 4A–C**; **Supplementary Figures 3A, B**; **Supplementary Table 2**). For each study, we scored the following parameters: 1) whether a full gating strategy was reported; 2) the total cells collected per sample for flow cytometry; 3) the total live cell counts during flow cytometry sample collection; 4) if the total immune cell subset percentage was calculated directly from the live cell count reported or if it was derived from another gate (i.e., if the immune cell subset percentage reported was derived from the CD45^+^ gate or from the live/dead gate); and 5) whether the methods used to determine cell counts and/or MFI readings were documented. Of the 58 studies reviewed, 35 [60.3%] included a full gating strategy (**Figure 4A**; **Supplementary Table 2**) (27, 30, 32–34, 36, 37, 41, 43–45, 47, 50, 51, 54, 55, 58, 60–64, 67, 71–79, 81, 82, 84), while 20 [34.5%] reported a partial gating strategy (28, 29, 35, 38, 40, 42, 46, 48, 49, 52, 53, 56, 57, 59, 66, 68–70, 80, 83)., and 3 [5.17%] did not include this information (31, 39, 65). When documented, the flow antibody clone and gating strategy were reported (44/58 studies; 75.9%) (**Supplementary Table 2**) (27, 29–33, 35–40, 42, 43, 45–55, 57, 58, 60–62, 66–69, 74–80, 82–84). Most studies used similar clones to identify specific immune cell subsets (**Supplementary Table 2**).

On reporting the total cells collected per sample during flow cytometry collection (**Figure 4B**; **Supplementary Table 2**), only 16/58 [27.6%] of studies provided this data (30, 36, 37, 41, 44, 47, 52, 57, 58, 61, 63, 67, 71, 73, 74, 82). Total live cell counts collected during flow cytometry (**Supplementary Figure 3A**; **Supplementary Table 2**) were only reported in 9/58 [15.5%] of the studies reviewed (30, 37, 57, 58, 61, 67, 71, 73, 74). Out of the 58 selected studies, only 12/58 [20.7%] expressed their results in terms of total immune cell subset percentages derived from live cells only (**Figure 4C**; **Supplementary Table 2**) and not from another gate (such as deriving from the CD45^+^ gate) (35, 37, 43, 47, 52, 58, 67, 68, 71, 72, 74, 76). Lastly, 35/58 [60.3%] of studies (**Supplementary Figure 3B**; **Supplementary Table 2**) reported methods on how cell counts and/or MFI readings were recorded (27, 28, 30, 32, 33, 35, 37, 39, 41–44, 49–52, 54, 55, 57–59, 61–64, 67, 70–77, 82). Overall, the cytometer/FACS sorter used and the reporting of total cells collected, total live cells, and immune cell subset percentages were not standardized in the 58 studies examined.

### 3.4 Reported immune cell counts from WT/control homogenized mouse brain are highly variable between studies

Across all the studies, we then examined the total immune cell counts reported in WT/control mouse models, as detected by flow cytometry immunophenotyping and/or by FACS (**Supplementary Table 3**). As described in the Methods and **Supplementary Table 3**, studies were selected based on their reporting of a wide variety of immune cell subsets. These included T cell subsets/memory T cells (naïve-like, central memory [T_CM_], effector memory [T_EM_], and effector memory re-expressing CD45RA [T_EMRA_]), natural killer cells (NK cells), dendritic cells (DCs), B cells, monocytes, macrophages (M1 [predominately CD86^+^] and M2 [predominately CD163^+^] phenotypes), TMEM119^+^ microglia, and neutrophils. Of note, the M1/M2 nomenclature for macrophages is currently being updated in the immunology field (88).

**Figure 5:**
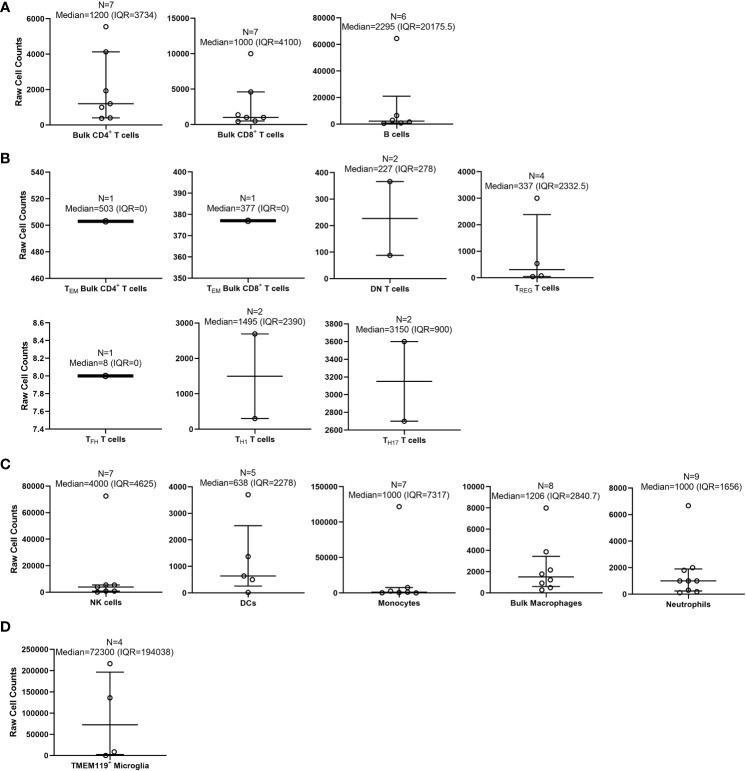
Calculated medians with interquartile ranges (IQRs) of immune cells quantified by flow cytometry within wild-type/control mouse brains. Medians with IQRs (defined as: 75^th^ percentile upper quartile [Q3] – 25^th^ percentile lower quartile [Q1]) of immune cell subset counts found with wild-type (WT)/control homogenized mouse brains were calculated from data extrapolated from the 58 studies selected for inclusion in this systematic review. Immune cell subsets were organized as follows: **(A)** bulk adaptive immune cells; **(B)** specialized T cells; **(C)** innate immune cells; and **(D)** microglia. Total n-values (number of studies for each identified subset) are reported along with the median and IQR for each immune subset above each bar graph.

Many immune subsets were examined, but relatively few were reported in a sufficient number of studies to be able to calculate representative medians with IQR and means with SD (**Figure 5**; **Supplementary Table 3**). Immune cell subsets with at least two or more references to derive a median and mean cell count for WT/control mice included bulk CD4^+^ T cells (27–33), bulk CD8^+^ T cells (27, 28, 30, 34–37), double negative (DN) T cells (33, 38), regulatory T cells (T_REG_) (29, 39–42), T helper 1 cells (T_H1_) (27, 40), T helper 17 cells (T_H17_) (44, 45), NK cells (33, 41, 46, 47, 49–51), DCs (33, 52–55), B cells (33, 56–60), monocytes (61–67), bulk macrophages (64, 65, 68–72), TMEM119^+^ microglia (73–76), and neutrophils (46, 77–84). Immune cell subset counts that were derived from only one study, such as follicular T cells (T_FH_) (43) and T_EM_ bulk CD4^+^ and CD8^+^ T cells (30), were still included in this review as a representation of the possible median/average cell count for these subsets.

We first calculated the overall medians for each examined immune subset by collecting all of the “raw” total immune subset cell counts from each of the 58 studies (**Figure 5**). By doing this, we discovered that out of these studies, there were some that were outliers (outside of the IQR) that appear to have heavily altered the overall total immune cell counts determined by flow cytometry (27, 33, 36, 41, 47, 53, 58, 61, 69, 72, 76, 79). Out of all the immune cell subsets from the 58 studies, the highest median was TMEM119^+^ microglia (72,300 [IQR=194,038]; n = 4 studies) (73–76). The lowest median that was calculated was T_FH_ T cells (8 [IQR=0]; n = 1 study) (43). Memory T cell subsets found within both CD4^+^ and CD8^+^ T cells were not reported as an immune cell subset in any study. Similarly, T helper 2 cells (T_H2_) were not reported in any studies.

The 58 studies had highly variable ranges for each immune cell subset found within mouse brain, for which most of the SD calculated was greater than the means (**Supplementary Table 3**). As with the calculated medians, the highest mean cell count reported from the 58 studies was TMEM119^+^ microglia (90,323 ± 104,555; n = 4 studies) (73–76). The lowest mean cell counts reported were for T_FH_ T cells (8 ± 0; n = 1 study) (43). Subsequently, we also calculated the means of total overall cell counts collected per sample, as reported in the 58 studies reviewed (**Supplementary Table 3**). Although not as variable as the immune cell subset counts, the total overall cell counts collected by flow cytometry ranged from 1 x 10^4^ to ~3 x 10^6^ cells (**Supplementary Table 3**). In some studies, the actual number of cells collected by flow cytometry was not specified, but the total cell counts could be extrapolated from data given in the main text, figures, and/or **Supplementary Materials**. Overall, we conclude the immune cell subset counts collected by flow cytometry immunophenotyping are highly variable between studies, potentially due to the processing method, technical skills, and experience of the researcher. Moreover, there are currently insufficient data on specific T cell subsets/memory subsets and specific macrophage phenotype counts within the WT mouse brain.

### 3.5 Standardizing immune cell counts and percentages within WT/control mouse brain

**Figure 6:**
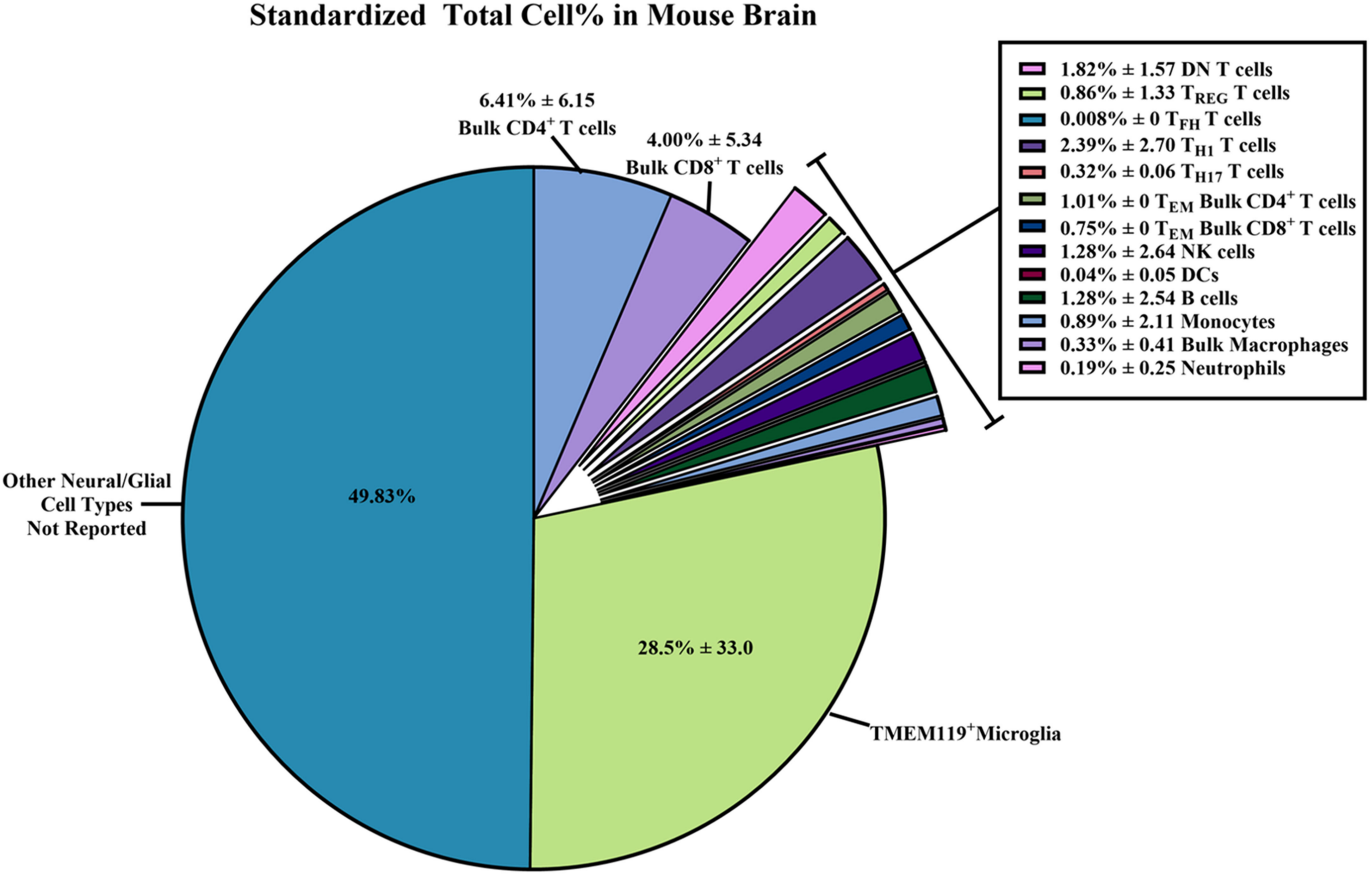
Standardized total cell percentages of immune cells quantified by flow cytometry within wild-type/control mouse brains. Estimated percentages of immune cell subset counts found within wild-type (WT)/control homogenized mouse brains were calculated from data extrapolated from the 58 studies selected for inclusion in this systematic review. The equations used to determine the standardized cell counts can be found in the Methods section. Briefly, “raw” total immune cell subset count and “raw” total cell count collected via flow cytometry were standardized assuming 1 x 10^5^ total cells were collected. Results were reported as combined means of percentages with standard deviations ( ± SD) from the standardized totals from each study, assuming 1 x 10^5^ total cells were collected.

We devised a method to standardize the counts of immune cells and subset percentages within WT/control mouse brains in each study (**Figure 6**; **Supplementary Table 3**). In flow cytometry, cell type percentages should be reported as values from the total number of cells collected (on average 1 x 10^5^ cells) rather than from a sub-partition within the gating strategy (26, 89). Thus, based on the assumption that the total cells collected per sample was 1 x 10^5^ cells, we were able to estimate a standardized total immune cell subset count for the data provided in each study.

From these standardized total immune cell subset counts, we were able to determine the proportion of immune cell subsets within the brain by simply dividing the standardized count by 1 x 10^5^ total cells collected to obtain percentages (**Figure 6**; **Supplementary Table 3**). The immune subset with the highest total cell percentages that were found within WT/control mouse brains was TMEM119^+^ microglia (28.5% ± 33.0). As for non-neural/glial specific immune cells, bulk CD4^+^ T cells (6.41% ± 6.15) and bulk CD8^+^ T cells (4.00% ± 5.34) were most often counted within mouse brains compared to other adaptive/innate immune cells. Overall, we were able to calculate the average percentage of immune cells found within WT/control mouse brains from the 58 selected studies. Hence, we are able to report a more reliable estimate of the immune cell composition within the mouse brain despite the wide SD.

### 3.6 Evaluating risk of bias of all included studies

**Figure 7:**
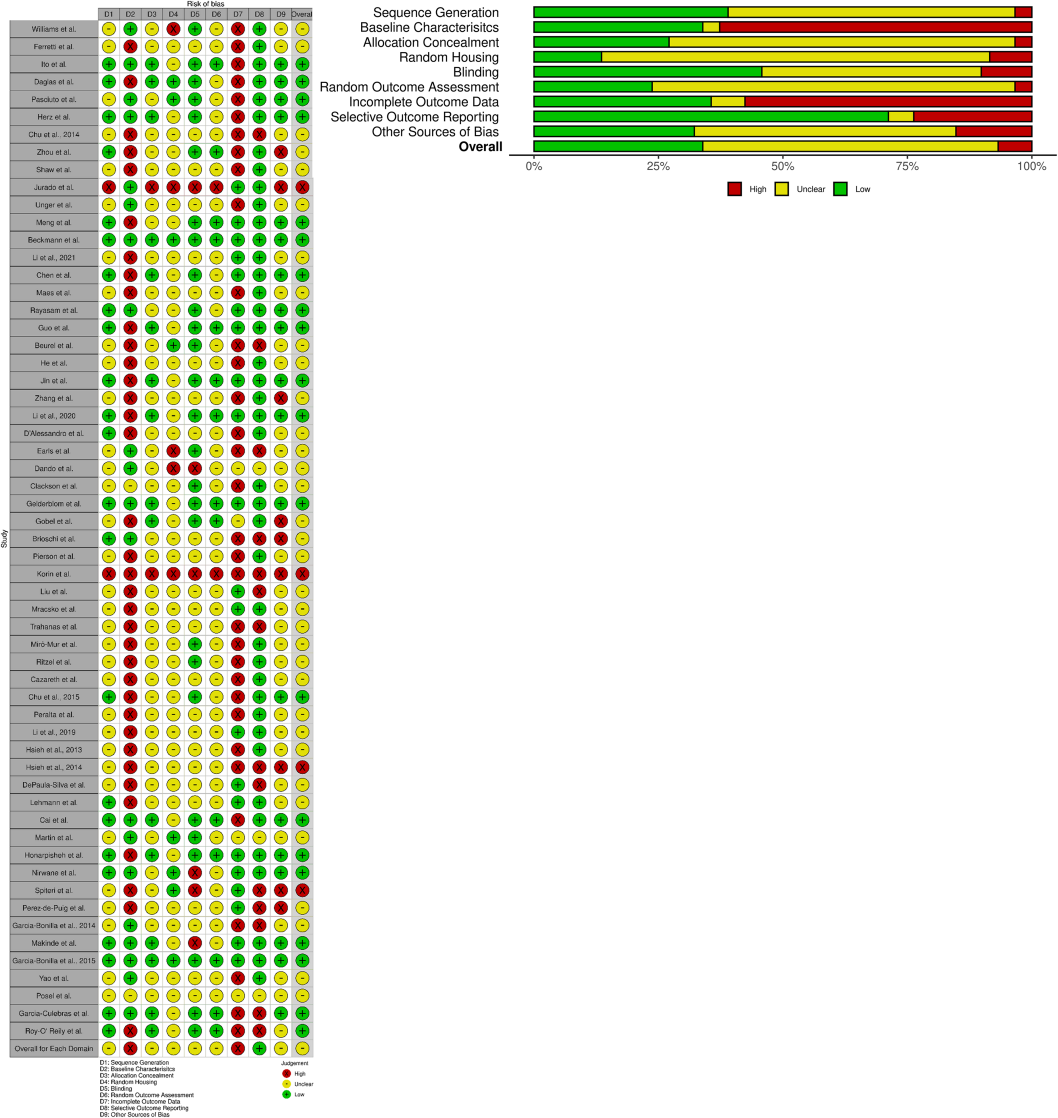
SYRCLE’s risk of bias: Tools for animal studies summarized for each study. Both the summary graph and “stop-light” figure were created via the *robvis* R package and Shiny web app (25). The criteria are based upon the Systematic Review Centre for Laboratory animal Experimentation (SYRCLE) methodology to detect bias (24). All 58 studies selected were reviewed and analyzed using SYRCLE criteria domains: D1: Sequence Generation; D2: Baseline Characteristics; D3: Allocation Concealment; D4: Random Housing; D5: Blinding; D6: Random Outcome Assessment; D7: Incomplete Outcome Data; D8: Selective Outcome Reporting; and D9: Other Sources of Bias. Red: High Bias; Yellow: “Unclear” Bias; Green: Low Bias.

As per the PRISMA and Cochrane criteria for systematic reviews, it is important to evaluate the risk of bias for all the cited studies (20, 21, 24, 90). Here, we utilize the SYRCLE’s risk of bias tools for animal studies (24) to create a summary graph and “stop-light” figure (**Figure 7**) to highlight the overall bias of each study assessed within the following domains: D1: Sequence Generation (randomization methods used to choose animals for comparable groups); D2: Baseline Characteristics (full description of animal characteristics from all comparable groups); D3: Allocation Concealment (methods used to conceal how animals are distributed to researchers, i.e., using a coding method for each animal); D4: Random Housing (housing all animal groups randomly within the animal room); D5: Blinding (blinding methods used on researchers, such as blinding the knowledge of intervention or transgenic model used and blinding the outcome assessors); D6: Random Outcome Assessment (methods on how the animals were selected at random for outcome assessment); D7: Incomplete Outcome Data (description of the completeness of the data outcome, i.e., stating if data were excluded or if animals were removed from the study at any point); D8: Selective Outcome Reporting (the completeness of the study protocols); D9: Other Sources of Bias (examples include confounders, contamination problems, analysis errors, and design-specific risk of bias, etc.).

The overall bias of all the selected studies was deemed predominantly “unclear” (~over 50%) due to lack of reporting on specific data/methodology required to pass the “high” or “low” bias questionnaire in each study (**Figure 7**). The most biased domains (~over 50% high bias scoring) from the selected studies were Baseline Characteristics (i.e., it was largely unclear how sex, age, weight, or other baseline characteristics or confounders were adjusted for in each analysis) and Incomplete Outcome Data (i.e., it was generally unclear whether all animals were included in each analysis and, if not, whether there was any report on why they were missing outcome data or how that missing data influenced the study). The lowest biased domain (~over 75% low bias scoring) was Selective Outcome Reporting (i.e., whether the results reported reflected the methods described in the selected studies). For further clarification of the methods used in order to clear up bias reporting, we contacted all 58 corresponding authors to request more information (all names and affiliations of the authors who responded are included in the Acknowledgements section). Overall, the reported bias from all the included studies was largely considered to be “unclear” due to the lack of reporting and transparency in the methods and results described. As such, this could be a possible reason for the high variability in the immune cell counts that were reported across multiple studies. Reliable reporting and including confounding factors within experimental procedures/data analysis is necessary for the meta-analysis of immunophenotypes found within the mouse brain.

## 4 Discussion and recommendations

The prominent role of the innate and acquired immune system in brain health and neurologic and age-associated neurodegenerative disorders has become increasingly apparent. In part, this has been driven by the immunologic role of several variant gene discoveries, including triggering receptor expressed on myeloid cells 2 (*TREM2*) in Alzheimer’s disease, granulin (*GRN*) in frontotemporal dementia, and leucine-rich repeat kinase 2 (*LRRK2*) in Parkinson’s disease (91–94). Despite this burgeoning interest in neuroimmunology and the many published studies, results from flow cytometry immunophenotyping from homogenized mouse brain are highly variable. Although this does not invalidate ‘within study’ comparisons of specific immune subsets, such variability is a challenge for reproducibility, meta-analysis across studies, and interpretation (27–84). Reliable data on residential and infiltrating immune cells within WT/control mouse brains would be of benefit (1–10). One step toward that goal is standardized reporting of flow cytometry methods and results and this being required to become a prerequisite for peer-reviewed publications. In this systematic review, we demonstrate most studies that apply flow cytometry methods to neurology and neuroimmunology, specifically to homogenized mouse brain, share little to no consensus on methods, analysis, or results. Here, we summarize our findings and produce a series of recommendations for future studies (**Table 1**).

**Table 1:** Minimum recommendations and sources for standards for future mouse brain flow cytometry reports (4, 5, 13, 15, 26, 89, 95–101).

Criteria	Recommendations and Notes
Mouse Strain	• Report background mouse strain being used as different mouse strains have different immune backgrounds and responses. • Ensure a WT or a non-treated mouse is included and housed in the same vivarium as transgenic/treated mice in order to enable cross-study comparison. • Report specific details of the vivarium and the caging conditions used. • DO NOT switch mouse strains in the middle of an experiment as immune profiles will vary drastically. • Ensure genetic, phenotypic, and supplier information is provided for each mouse strain used.
Mouse Age	• Report the ages of the mice used for the study as immune profiles are altered by age. • For a more naïve, younger immune cell population, it is best to utilize mice aged ~3 months. • For a more mature, older immune cell population, it is best to utilize mice aged ~18 months. • Age can affect neurological phenotype and behavior; thus, it needs to be included as a confounder.
Mouse Sex	• Report the sex of the mice used, as sex contributes towards different immune responses. • Ensure that the same sex of mice is used throughout all experiments within the study unless the study is focused on sex differences. • Use power analysis based upon sex-specific variances to estimate male to female ratios.
Perfusion Techniques	• Perfusion is recommended before brain homogenization to avoid circulating blood/immune cell contamination. • Use cold Hank’s balanced salt solution (HBSS) with 10% serum (heat-inactive fetal bovine serum [FBS], mouse serum, etc.) via cardiac perfusion to ensure residential immune cells and neurological cells remain intact and viable for flow cytometry. • Use of 4% paraformaldehyde (PFA) in any perfusion mixture will affect flow cytometry staining and should only be included AFTER staining for surface antigens.
Brain Dissociation and Cell Isolation Techniques	• Mechanical homogenization (Dounce tissue grinder, bead beating, sonication, etc.), enzymatic homogenization (collagenase, DNase I, trypsin-EDTA, etc.), or the combination of both can alter the phenotypes of immune cells; thus, it is important to describe the technique used. • Use one or more cell strainers (preferably using a 100 μm filter first, then a 70 μm filter) in order to remove as much excess dead cells, fats, and myelin, etc., as possible as these components will alter flow cytometry collection and readings. • Use an isotonic Percoll gradient in order to isolate immune cells and other cells of interest. At minimum, a 37% isotonic Percoll gradient is required to remove all myelin (top layer) and isolate all brain cells (bottom layer/cell pellet). • A 30%/37%/70% isotonic Percoll solution is also recommended if microglia isolation is the only objective.
Flow Cytometer/Sorter Equipment and Analysis Software Reporting	• Report the exact specifications of the flow cytometer/sorter used in the study. • DO NOT switch between cytometers/sorters throughout experimentation as different models can produce different results. Ideally, calibration and optimization of the machine should be performed daily to ensure accurate results. • Report flow cytometry antibody information, such as clone ID, fluorophore-conjugated, targets, and source, etc. • Analysis software and version number should be reported.
Flow Cytometry Result Reporting	• Include a representation of the entire gating strategy (from forward scatter/side scatter to single cells, to live dead, to beginning of gating strategy, and beyond) for each type of experiment conducted. These figures should come from a WT/non-treated control mouse. • State that the results provided are standardized and comparable to multiple flow cytometry controls, such as unstained, FMO, and single-color controls. At a minimum, either unstained and/or FMO control gating should be included in the report. • Provide cell counts and cell percentages for each gate reported. • Histograms should include unstained and FMO results along with the subjects’ data. Indicate MFI readings for each histogram included. • Report exact or estimated total cells per sample collected by flow cytometry either as a unified cell count (i.e., all samples had 1 x 10^5^ cells collected during flow cytometry) or for each individual sample. Report the total number of CD45^+^ cells (all infiltrating/residential immune cells) and CD45^-/lo^ cells (all microglia and other glial cells) when counting immune cells within mouse brain. • Use a live/dead dye to separate living cells for counts. Include the total amount of live cells counted on average and/or from each sample. • Report cell subsets as either total cell counts or by percentages derived from either live cells or from total cells collected. • Methods used to describe how cell counts were reported (by use of flow cytometry cell counting beads, counts provided by the machine, counts calculated, etc.) or how MFI’s were calculated are recommended. • Deposit raw data, such as FCS files, in a database such as the FlowRepository (http://flowrepository.org/) after full analysis is completed.

We retrieved 58 neurological/neuroimmunology studies that utilized flow cytometry to identify or sort multiple immune cell subsets from WT/control mouse brains, which were generally compared within the study results to an experimental mouse model (27–84). We compared mouse strains, perfusion, and tissue processing methods and noted that the age of mice and methods for tissue homogenization are variable (96–98, 102). Vivarium conditions, such as group housing within ventilated racks in a pathogen-free barrier facility versus more conventional non-barrier non-ventilated caging, were seldom documented. Corresponding authors from 10/58 studies (17.2%) indicated that the majority of studies utilized a barrier facility with HEPA-filtered air, where each cage was individually ventilated and had sterilized bedding and chow (27, 39, 45, 51, 52, 58, 62, 73, 75, 76). Housing conditions, age, and sex influence the immune cell subsets that can be identified by flow cytometry (95–98) and should be carefully considered, documented in experimental protocols, and adjusted for as a covariate in subsequent analyses. When deciding on immune cell isolation methods for mouse brains, both mechanical homogenization or/and enzymatic tissue digestion are appropriate. Nevertheless, each approach has pros and cons on immune cell retrieval and phenotypic expression and, depending on the specific research question, must be carefully considered (102–104).

Once the mouse lines and homogenization/isolation methods were analyzed, we compared flow cytometry techniques and data reporting across the 58 studies. Cytometers/FACS sorters come in a variety of makes and models but essentially perform the same function and should be calibrated using universal standards (89, 99, 105). Many useful guidelines exist for reporting and include the Minimum Information about a Flow Cytometry Experiment (MIFlowCyt) criteria (26, 89, 99, 106). These recommend researchers present flow cytometry data and methods by reporting: 1) the sample/specimen used for experiments; 2) how the samples were treated (storage, processing, and staining, etc.); 3) what reagents were used and which antibody clones; 4) what controls were used (unstained controls, FMOs, and single-color controls, etc.) with a demonstration of the full gating strategy for each panel; 5) what instrument was used and details about it; 6) how many total cells and/or live cells were collected in each sample (either exact cell counts or overall estimated counts); 7) what analysis software was used and how compensation was calculated. Although these guidelines do recommend reporting the total cells and/or live cells collected in each sample, this can be misleading for brain homogenate studies that use different tissue dissociation and cell isolation techniques. For example, a 30%/70% Percoll gradient solution will preferentially isolate immune cells, whereas a 30% solution will isolate immune cells and other residential brain cells (neurons and astrocytes, etc.). Hence, it would be beneficial to report all CD45^+^ (infiltrating/residential immune cells within the brain) and CD45^-/lo^ (microglia and other glial/brain cells). Better documentation would enable replication and more reliable and accurate results and enable subsequent meta-analysis on the immunophenotyping of neurogenerative mouse models.

In the 58 selected studies, we next examined the median and average count and percentages of immune cell subsets reported from WT/control mouse brains. The immune cell subsets selected (**Figures 5**, **6**; **Supplementary Table 3**) consisted of brain/CNS-only residential immune cells (microglia) and immune cells considered to be both residential and peripheral (i.e., T cells, B cells, macrophages, and NK cells) (1–10). As expected, of all the immune subsets examined, microglia are the most populous immune cells within WT/control mouse brains (15, 74–76). For microglia markers, we searched for publications that showed expression of TMEM119, which is expressed in more stable, non-reactive microglia (107–110). Traditional methods of detecting microglia by flow cytometry use CD11b^+^ CD45^lo/-^ phenotyping. However, we favored microglia-specific markers such as TMEM119 as these differentiate microglia from other phagocytic cell types.

For other immune cell subsets, T cells make up the second most abundant immune cell within WT/control mouse brains, with more bulk CD4^+^ T cells (27–37). In the brain, it is likely that both CD4^+^/CD8^+^ T cells are comprised of resident memory T cells (T_RM_) and might be classified as an even more specialized subset (i.e., T_CM_, T_EM_, and T_EMRA_) (111–113). For the innate immune cell populations (excluding microglia), NK cells were observed more frequently in WT/control mouse brains than other innate immune cells (33, 46–51). Many studies report NK cells as most abundant within the brain’s parenchyma and more often than other innate immune populations (excluding microglia) or adaptive immune cells (T cell and B cells) (114–116). NK cells were the most reported innate immune cell subset within WT/control mouse brains in the 58 studies reviewed, but there were still more bulk CD4^+^/CD8^+^ T cell counts reported. We cautiously included neutrophils in our systematic review (46, 77–84). These polynucleated cells are challenging to detect with flow cytometry as neutrophil extracellular traps (NETs) cause them to be extremely “sticky”, to bind onto each other, and to bind non-specifically to flow antibodies (causing false positives) (117–120). The variability in nomenclature/targets to identify neutrophils, their short life span, and sensitivity toward purification methods are additional limitations (117–120).

The presence of less abundant immune cell subsets found within the brain or infiltrating the brain, including specific CD4^+^ T cell subsets, T cell memory subsets, and DCs, was also assessed. Unpredictably, it appears that T_H1_ T cells are more abundant in WT/control mouse brains than T_H2_ T cells (not reported in the 58 selected studies) (27, 40). Conventionally, the T_H1_/T_H2_ ratio is used to determine whether an individual has a bacterial/viral infection (higher ratios are indicative of greater infection), although higher ratios of these subsets are also found in aged subjects, once again highlighting the importance of defining age in studies of immunity (121). None of the 58 studies reported specific T cell memory subsets besides bulk T_EM_ CD4^+^/CD8^+^ T cells (30) within WT/control mice. Given the importance of specific T cell memory subsets to overall immunity, future flow cytometry analysis of the brain may benefit from their inclusion. Surprisingly, a few reports listed migratory/residential DCs within WT/control mouse brains, albeit at extremely low levels (33, 52–55) as microglia are thought to mediate brain immune surveillance (122–124).

Our systematic review has some limitations. Our analysis and database search were not automated to update figures from more recent research (125, 126), and potentially, this may be considered a selection bias. The keyword searches we conducted for each immune cell subset have been reported in our methods, but different variations of these names or use of other abbreviations when searching could alter what literature is identified in each database; thus, this could also be considered some level of bias. As several of the selected studies failed to report sufficient details within their main text, figures, or **Supplementary Materials**, whenever possible, data were extrapolated. We attempted to avoid any author bias or incorrect statements as two independent reviewers assessed all the manuscripts. The original authors were also contacted when further clarifications were required.

Bias of each individual study was reported as per the guidelines created by multiple organizations that review and conduct systematic reviews (18–24). With a majority of the studies examined, very few were able to clearly state if there was any bias or not within the experimental design. As such, we deemed most of the studies as “unclear” bias due to the lack actual reporting on specific data/methodology that could pass as “high” or “low” bias. This can be very problematic as, for example, the Baseline Characteristics category of bias was unclear or high in a majority of the 58 studies. Not including mouse baseline characteristics such as sex, age, weight, and housing conditions, etc., as confounders of experimentation is extremely problematic and can lead to high bias due to the dramatic effect of these factors affecting the immune system of each individual mouse model used. As such, factors such as these can heavily affect the results of flow cytometry testing.

We recommend that supplementary data should include all raw cell counts and document which data are used in the main figures and text to provide more transparency and enable reproducibility in flow cytometry experiments. Overall, there was tremendous variability in the immune cell subset counts in the 58 reviewed studies, such that the SD often exceeded the mean estimates. Hence, medians with IQRs have been provided throughout this review. This could be due to a wide variety of reasons, such as the technical skills/experience of the researcher, reporting bias, and unconsidered confounding factors. There are also methods reporting variability that can contribute toward immune cell count variance across studies, such as the following: mouse strain/age/sex, perfusion, and brain tissue processing techniques conducted, flow cytometer used, fluorescent antibodies used, and gating strategy used. Although heterogeneity in instrumentation and procedures can be unavoidable at some points and is dependent upon the facility where the research is conducted, it would be helpful to standardize some aspects of how immunophenotyping and cell counts are reported. While within-study comparisons are still valid, researchers should report more robust information about mouse parameters, brain tissue processing, and flow cytometry procedures in order to be replicated in the field. It would be insightful to compare flow cytometry results across studies, not only within them. As such, in our analysis, we have included a series of recommendations to aid the interpretation of results, reproducibility, and meta-analysis (**Table 1**). Adherence to reporting guidelines will ultimately improve our understanding of the dynamic role of immunity in mouse brain.

## Data availability statement

The datasets presented in this study can be found in online repositories. The names of the repository/repositories and accession number(s) can be found in the article/**Supplementary Material**.

## Author contributions

RS: Conceptualization, Data curation, Formal Analysis, Investigation, Methodology, Software, Visualization, Writing – original draft, Writing – review & editing. DG: Data curation, Formal Analysis, Methodology, Writing – review & editing. MF: Conceptualization, Funding acquisition, Investigation, Project administration, Resources, Supervision, Writing – original draft, Writing – review & editing.

## References

[1] Dawson TM Golde TE Lagier-Tourenne C . Animal models of neurodegenerative diseases. Nat Neurosci (2018) 21:1370–9. doi: 10.1038/s41593-018-0236-8 PMC6615039 30250265

[2] Chesselet M-F Carmichael ST . Animal models of neurological disorders. Neurotherapeutics (2012) 9:241–4. doi: 10.1007/s13311-012-0118-9 PMC3337025 22460561

[3] Hafezparast M Ahmad-Annuar A Wood NW Tabrizi SJ Fisher EM . Mouse models for neurological disease. Lancet Neurol (2002) 1:215–24. doi: 10.1016/S1474-4422(02)00100-X 12849454

[4] Croxford JL Miyake S . “Animal models for the study of neuroimmunological disease.,”. In: Neuroimmunological Diseases. Tokyo: Springer Japan (2016). p. 33–54. doi: 10.1007/978-4-431-55594-0_3

[5] da Silva APB Silva RBM Goi LDS Molina RD MaChado DC Sato DK . Experimental models of neuroimmunological disorders: A review. Front Neurol (2020) 11:389. doi: 10.3389/fneur.2020.00389 32477252 PMC7235321

[6] Grotemeyer A McFleder RL Wu J Wischhusen J Ip CW . Neuroinflammation in Parkinson’s disease – putative pathomechanisms and targets for disease-modification. Front Immunol (2022) 13:878771. doi: 10.3389/fimmu.2022.878771 35663989 PMC9158130

[7] Saito T Saido TC . Neuroinflammation in mouse models of Alzheimer’s disease. Clin Exp Neuroimmunol (2018) 9:211–8. doi: 10.1111/cen3.12475 PMC6282739 30546389

[8] Passaro AP Lebos AL Yao Y Stice SL . Immune response in neurological pathology: emerging role of central and peripheral immune crosstalk. Front Immunol (2021) 12:676621. doi: 10.3389/fimmu.2021.676621 34177918 PMC8222736

[9] Tan EK Chao YX West A Chan LL Poewe W Jankovic J . Parkinson disease and the immune system — associations, mechanisms and therapeutics. Nat Rev Neurol (2020) 16:303–18. doi: 10.1038/s41582-020-0344-4 32332985

[10] Ma Q Xing C Long W Wang HY Liu Q Wang R-F . Impact of microbiota on central nervous system and neurological diseases: the gut-brain axis. J Neuroinflamm (2019) 16:53. doi: 10.1186/s12974-019-1434-3 PMC6397457 30823925

[11] Hu P Zhang W Xin H Deng G . Single cell isolation and analysis. Front Cell Dev Biol (2016) 4:116. doi: 10.3389/fcell.2016.00116 27826548 PMC5078503

[12] Lanz TV Pröbstel A-K Mildenberger I Platten M Schirmer L . Single-cell high-throughput technologies in cerebrospinal fluid research and diagnostics. Front Immunol (2019) 10:1302. doi: 10.3389/fimmu.2019.01302 31244848 PMC6579921

[13] Drescher H Weiskirchen S Weiskirchen R . Flow cytometry: A blessing and a curse. Biomedicines (2021) 9:1613. doi: 10.3390/biomedicines9111613 34829841 PMC8615642

[14] de Graaf MT de Jongste AHC Kraan J Boonstra JG Smitt PAES Gratama JW . Flow cytometric characterization of cerebrospinal fluid cells. Cytometry B Clin Cytom (2011) 80B:271–81. doi: 10.1002/cyto.b.20603 21567940

[15] Martin D Xu J Porretta C Nichols CD . Neurocytometry: flow cytometric sorting of specific neuronal populations from human and rodent brain. ACS Chem Neurosci (2017) 8:356–67. doi: 10.1021/acschemneuro.6b00374 PMC6385603 28135061

[16] Brioschi S d’Errico P Amann LS Janova H Wojcik SM Meyer-Luehmann M . Detection of synaptic proteins in microglia by flow cytometry. Front Mol Neurosci (2020) 13:149. doi: 10.3389/fnmol.2020.00149 33132837 PMC7550663

[17] Mrdjen D Pavlovic A Hartmann FJ Schreiner B Utz SG Leung BP . High-dimensional single-cell mapping of central nervous system immune cells reveals distinct myeloid subsets in health, aging, and disease. Immunity (2018) 48:380–95.e6. doi: 10.1016/j.immuni.2018.01.011 29426702

[18] Kim H Sefcik JS Bradway C . Characteristics of qualitative descriptive studies: A systematic review. Res Nurs Health (2017) 40:23–42. doi: 10.1002/nur.21768 27686751 PMC5225027

[19] Hooijmans CR IntHout J Ritskes-Hoitinga M Rovers MM . Meta-analyses of animal studies: an introduction of a valuable instrument to further improve healthcare. ILAR J (2014) 55:418–26. doi: 10.1093/ilar/ilu042 PMC4276598 25541544

[20] Vesterinen HM Sena ES Egan KJ Hirst TC Churolov L Currie GL . Meta-analysis of data from animal studies: A practical guide. J Neurosci Methods (2014) 221:92–102. doi: 10.1016/j.jneumeth.2013.09.010 24099992

[21] Moher D Liberati A Tetzlaff J Altman DG . Preferred reporting items for systematic reviews and meta-analyses: the PRISMA statement. PloS Med (2009) 6:e1000097. doi: 10.1371/journal.pmed.1000097 19621072 PMC2707599

[22] Clarke M Oxman A Paulsen E Higgins J Green S . Guide to the contents of a Cochrane Methodology protocol and review. Cochrane Collaboration (2020) 5:1.0.

[23] Munn Z Stern C Aromataris E Lockwood C Jordan Z . What kind of systematic review should I conduct? A proposed typology and guidance for systematic reviewers in the medical and health sciences. BMC Med Res Methodol (2018) 18:5. doi: 10.1186/s12874-017-0468-4 29316881 PMC5761190

[24] Hooijmans CR Rovers MM de Vries RB Leenaars M Ritskes-Hoitinga M Langendam MW . SYRCLE’s risk of bias tool for animal studies. BMC Med Res Methodol (2014) 14:43. doi: 10.1186/1471-2288-14-43 24667063 PMC4230647

[25] McGuinness LA Higgins JPT . Risk-of-bias VISualization (robvis): An R package and Shiny web app for visualizing risk-of-bias assessments. Res Synth Methods (2021) 12:55–61. doi: 10.1002/jrsm.1411 32336025

[26] Cossarizza A Chang H Radbruch A Abrignani S Addo R Akdis M . Guidelines for the use of flow cytometry and cell sorting in immunological studies (third edition). Eur J Immunol (2021) 51:2708–3145. doi: 10.1002/eji.202170126 34910301 PMC11115438

[27] Williams GP Schonhoff AM Jurkuvenaite A Gallups NJ Standaert DG Harms AS . CD4 T cells mediate brain inflammation and neurodegeneration in a mouse model of Parkinson’s disease. Brain (2021) 144:2047–59. doi: 10.1093/brain/awab103 PMC8370411 33704423

[28] Ferretti MT Merlini M Späni C Gericke C Schweizer N Enzmann G . T-cell brain infiltration and immature antigen-presenting cells in transgenic models of Alzheimer’s disease-like cerebral amyloidosis. Brain Behav Immun (2016) 54:211–25. doi: 10.1016/j.bbi.2016.02.009 26872418

[29] Ito M Komai K Mise-Omata S Iizuka-Koga M Noguchi Y Kondo T . Brain regulatory T cells suppress astrogliosis and potentiate neurological recovery. Nature (2019) 565:246–50. doi: 10.1038/s41586-018-0824-5 30602786

[30] Daglas M Draxler DF Ho H McCutcheon F Galle A Au AE . Activated CD8+ T cells cause long-term neurological impairment after traumatic brain injury in mice. Cell Rep (2019) 29:1178–91.e6. doi: 10.1016/j.celrep.2019.09.046 31665632

[31] Pasciuto E Burton OT Roca CP Lagou V Rajan WD Theys T . Microglia require CD4 T cells to complete the fetal-to-adult transition. Cell (2020) 182:625–40.e24. doi: 10.1016/j.cell.2020.06.026 32702313 PMC7427333

[32] Herz J Köster C Crasmöller M Abberger H Hansen W Felderhoff-Müser U . Peripheral T cell depletion by FTY720 exacerbates hypoxic-ischemic brain injury in neonatal mice. Front Immunol (2018) 9:1696. doi: 10.3389/fimmu.2018.01696 30127782 PMC6087766

[33] Chu HX Kim HA Lee S Moore JP Chan CT Vinh A . Immune cell infiltration in Malignant middle cerebral artery infarction: comparison with transient cerebral ischemia. J Cereb Blood Flow Metab (2014) 34:450–9. doi: 10.1038/jcbfm.2013.217 PMC3948121 24326388

[34] Zhou Y Wang X Tang D Li Y Jiao Y Gan Y . IL-2mAb reduces demyelination after focal cerebral ischemia by suppressing CD8 ^+^ T cells. CNS Neurosci Ther (2019) 25:532–43. doi: 10.1111/cns.13084 PMC6488908 30444079

[35] Shaw TN Stewart-Hutchinson PJ Strangward P Dandamudi DB Coles JA Villegas-Mendez A . Perivascular arrest of CD8+ T cells is a signature of experimental cerebral malaria. PLoS Pathog (2015) 11:e1005210. doi: 10.1371/journal.ppat.1005210 26562533 PMC4643016

[36] Jurado KA Yockey LJ Wong PW Lee S Huttner AJ Iwasaki A . Antiviral CD8 T cells induce Zika-virus-associated paralysis in mice. Nat Microbiol (2017) 3:141–7. doi: 10.1038/s41564-017-0060-z PMC5780207 29158604

[37] Unger MS Li E Scharnagl L Poupardin R Altendorfer B Mrowetz H . CD8+ T-cells infiltrate Alzheimer’s disease brains and regulate neuronal- and synapse-related gene expression in APP-PS1 transgenic mice. Brain Behav Immun (2020) 89:67–86. doi: 10.1016/j.bbi.2020.05.070 32479993

[38] Meng H Zhao H Cao X Hao J Zhang H Liu Y . Double-negative T cells remarkably promote neuroinflammation after ischemic stroke. Proc Natl Acad Sci (2019) 116:5558–63. doi: 10.1073/pnas.1814394116 PMC6431175 30819895

[39] Beckmann L Obst S Labusek N Abberger H Köster C Klein-Hitpass L . Regulatory T cells contribute to sexual dimorphism in neonatal hypoxic-ischemic brain injury. Stroke (2022) 53:381–90. doi: 10.1161/STROKEAHA.121.037537 PMC8785522 34983246

[40] Li W Luo Y Xu H Ma Q Yao Q . Imbalance between T helper 1 and regulatory T cells plays a detrimental role in experimental Parkinson’s disease in mice. J Int Med Res (2021) 49:30006052199847. doi: 10.1177/0300060521998471 PMC8053775 33853440

[41] Chen C Chencheng Z Cuiying L Xiaokun G . Plasmacytoid dendritic cells protect against middle cerebral artery occlusion induced brain injury by priming regulatory T cells. Front Cell Neurosci (2020) 14:8. doi: 10.3389/fncel.2020.00008 32076400 PMC7006436

[42] Maes W Verschuere T Van Hoylandt A Boon L Van Gool S . Depletion of regulatory T cells in a mouse experimental glioma model through anti-CD25 treatment results in the infiltration of non-immunosuppressive myeloid cells in the brain. Clin Dev Immunol (2013) 2013:1–6. doi: 10.1155/2013/952469 PMC3655451 23710206

[43] Rayasam A Kijak JA Kissel L Choi YH Kim T Hsu M . CXCL13 expressed on inflamed cerebral blood vessels recruit IL-21 producing TFH cells to damage neurons following stroke. J Neuroinflamm (2022) 19:125. doi: 10.1186/s12974-022-02490-2 PMC9145182 35624463

[44] Guo Y Chen X Li D Liu H Ding Y Han R . PR-957 mediates neuroprotection by inhibiting Th17 differentiation and modulating cytokine production in a mouse model of ischaemic stroke. Clin Exp Immunol (2018) 193:194–206. doi: 10.1111/cei.13132 29603201 PMC6046491

[45] Beurel E Lowell JA Jope RS . Distinct characteristics of hippocampal pathogenic TH17 cells in a mouse model of depression. Brain Behav Immun (2018) 73:180–91. doi: 10.1016/j.bbi.2018.04.012 PMC6287768 29698707

[46] He H Geng T Chen P Wang M Hu J Kang L . NK cells promote neutrophil recruitment in the brain during sepsis-induced neuroinflammation. Sci Rep (2016) 6:27711. doi: 10.1038/srep27711 27270556 PMC4897692

[47] Jin W-N Shi K He W Sun J-H Van Kaer L Shi F-D . Neuroblast senescence in the aged brain augments natural killer cell cytotoxicity leading to impaired neurogenesis and cognition. Nat Neurosci (2021) 24:61–73. doi: 10.1038/s41593-020-00745-w 33257875

[48] Zhang Y Fung ITH Sankar P Chen X Robison LS Ye L . Depletion of NK cells improves cognitive function in the Alzheimer disease mouse model. J Immunol (2020) 205:502–10. doi: 10.4049/jimmunol.2000037 PMC7343613 32503894

[49] Li Z Li M Shi SX Yao N Cheng X Guo A . Brain transforms natural killer cells that exacerbate brain edema after intracerebral hemorrhage. J Exp Med (2020) 217:1–15. doi: 10.1084/jem.20200213 PMC7526480 32870258

[50] D’Alessandro G Antonangeli F Marrocco F Porzia A Lauro C Santoni A . Gut microbiota alterations affect glioma growth and innate immune cells involved in tumor immunosurveillance in mice. Eur J Immunol (2020) 50:705–11. doi: 10.1002/eji.201948354 PMC7216943 32034922

[51] Earls RH Menees KB Chung J Gutekunst C-A Lee HJ Hazim MG . NK cells clear α-synuclein and the depletion of NK cells exacerbates synuclein pathology in a mouse model of α-synucleinopathy. Proc Natl Acad Sci (2020) 117:1762–71. doi: 10.1073/pnas.1909110117 PMC6983411 31900358

[52] Dando SJ Naranjo Golborne C Chinnery HR Ruitenberg MJ McMenamin PG . A case of mistaken identity: CD11c-eYFP ^+^ cells in the normal mouse brain parenchyma and neural retina display the phenotype of microglia, not dendritic cells. Glia (2016) 64:1331–49. doi: 10.1002/glia.23005 27189804

[53] Clarkson BD Walker A Harris MG Rayasam A Hsu M Sandor M . CCR7 deficient inflammatory Dendritic Cells are retained in the Central Nervous System. Sci Rep (2017) 7:42856. doi: 10.1038/srep42856 28216674 PMC5316931

[54] Gelderblom M Gallizioli M Ludewig P Thom V Arunachalam P Rissiek B . IL-23 (Interleukin-23)–producing conventional dendritic cells control the detrimental IL-17 (Interleukin-17) response in stroke. Stroke (2018) 49:155–64. doi: 10.1161/STROKEAHA.117.019101 29212740

[55] Göbel K Pankratz S Asaridou C-M Herrmann AM Bittner S Merker M . Blood coagulation factor XII drives adaptive immunity during neuroinflammation via CD87-mediated modulation of dendritic cells. Nat Commun (2016) 7:11626. doi: 10.1038/ncomms11626 27188843 PMC4873982

[56] Brioschi S Wang W-L Peng V Wang M Shchukina I Greenberg ZJ . Heterogeneity of meningeal B cells reveals a lymphopoietic niche at the CNS borders. Sci (1979) (2021) 373:1–14. doi: 10.1126/science.abf9277 PMC8448524 34083450

[57] Pierson ER Stromnes IM Goverman JM . B cells promote induction of experimental autoimmune encephalomyelitis by facilitating reactivation of T cells in the central nervous system. J Immunol (2014) 192:929–39. doi: 10.4049/jimmunol.1302171 PMC3934009 24367024

[58] Korin B Ben-Shaanan TL Schiller M Dubovik T Azulay-Debby H Boshnak NT . High-dimensional, single-cell characterization of the brain’s immune compartment. Nat Neurosci (2017) 20:1300–9. doi: 10.1038/nn.4610 28758994

[59] Liu C Yang J Zhang C Geng X Zhao H . Remote ischemic conditioning reduced cerebral ischemic injury by modulating inflammatory responses and ERK activity in type 2 diabetic mice. Neurochem Int (2020) 135:104690. doi: 10.1016/j.neuint.2020.104690 31981607

[60] Mracsko E Javidi E Na S-Y Kahn A Liesz A Veltkamp R . Leukocyte invasion of the brain after experimental intracerebral hemorrhage in mice. Stroke (2014) 45:2107–14. doi: 10.1161/STROKEAHA.114.005801 24916913

[61] Trahanas DM Cuda CM Perlman H Schwulst SJ . Differential activation of infiltrating monocyte-derived cells after mild and severe traumatic brain injury. Shock (2015) 43:255–60. doi: 10.1097/SHK.0000000000000291 PMC4476068 26091024

[62] Miró-Mur F Pérez-de-Puig I Ferrer-Ferrer M Urra X Justicia C Chamorro A . Immature monocytes recruited to the ischemic mouse brain differentiate into macrophages with features of alternative activation. Brain Behav Immun (2016) 53:18–33. doi: 10.1016/j.bbi.2015.08.010 26275369

[63] Ritzel RM Patel AR Grenier JM Crapser J Verma R Jellison ER . Functional differences between microglia and monocytes after ischemic stroke. J Neuroinflamm (2015) 12:106. doi: 10.1186/s12974-015-0329-1 PMC4465481 26022493

[64] Cazareth J Guyon A Heurteaux C Chabry J Petit-Paitel A . Molecular and cellular neuroinflammatory status of mouse brain after systemic lipopolysaccharide challenge: importance of CCR2/CCL2 signaling. J Neuroinflamm (2014) 11:132. doi: 10.1186/1742-2094-11-132 PMC4237883 25065370

[65] Chu HX Broughton BRS Ah Kim H Lee S Drummond GR Sobey CG . Evidence that ly6C ^hi^ monocytes are protective in acute ischemic stroke by promoting M2 macrophage polarization. Stroke (2015) 46:1929–37. doi: 10.1161/STROKEAHA.115.009426 25999385

[66] Peralta Ramos JM Iribarren P Bousset L Melki R Baekelandt V van der Perren A . Peripheral inflammation regulates CNS immune surveillance through the recruitment of inflammatory monocytes upon systemic α-synuclein administration. Front Immunol (2019) 10:80. doi: 10.3389/fimmu.2019.00080 30761145 PMC6361759

[67] Li Q Lan X Han X Wang J . Expression of tmem119/sall1 and Ccr2/CD69 in FACS-sorted microglia- and monocyte/macrophage-enriched cell populations after intracerebral hemorrhage. Front Cell Neurosci (2019) 12:520. doi: 10.3389/fncel.2018.00520 30687011 PMC6333739

[68] Hsieh CL Kim CC Ryba BE Niemi EC Bando JK Locksley RM . Traumatic brain injury induces macrophage subsets in the brain. Eur J Immunol (2013) 43:2010–22. doi: 10.1002/eji.201243084 PMC4210355 23630120

[69] Hsieh CL Niemi EC Wang SH Lee CC Bingham D Zhang J . CCR2 deficiency impairs macrophage infiltration and improves cognitive function after traumatic brain injury. J Neurotrauma (2014) 31:1677–88. doi: 10.1089/neu.2013.3252 PMC4545982 24806994

[70] DePaula-Silva AB Gorbea C Doty DJ Libbey JE Sanchez JMS Hanak TJ . Differential transcriptional profiles identify microglial- and macrophage-specific gene markers expressed during virus-induced neuroinflammation. J Neuroinflamm (2019) 16:152. doi: 10.1186/s12974-019-1545-x PMC6642742 31325960

[71] Lehmann ML Cooper HA Maric D Herkenham M . Social defeat induces depressive-like states and microglial activation without involvement of peripheral macrophages. J Neuroinflamm (2016) 13:224. doi: 10.1186/s12974-016-0672-x PMC5007852 27581371

[72] Cai W Liu S Hu M Sun X Qiu W Zheng S . Post-stroke DHA treatment protects against acute ischemic brain injury by skewing macrophage polarity toward the M2 phenotype. Transl Stroke Res (2018) 9:669–80. doi: 10.1007/s12975-018-0662-7 30203370

[73] Martin E El-Behi M Fontaine B Delarasse C . Analysis of microglia and monocyte-derived macrophages from the central nervous system by flow cytometry. J Visualized Experiments (2017) 124. doi: 10.3791/55781 PMC5608497 28671658

[74] Honarpisheh P Lee J Banerjee A Blasco-Conesa MP Honarpisheh P d’Aigle J . Potential caveats of putative microglia-specific markers for assessment of age-related cerebrovascular neuroinflammation. J Neuroinflamm (2020) 17:366. doi: 10.1186/s12974-020-02019-5 PMC7709276 33261619

[75] Nirwane A Yao Y . SMAlow/undetectable pericytes differentiate into microglia- and macrophage-like cells in ischemic brain. Cell Mol Life Sci (2022) 79:264. doi: 10.1007/s00018-022-04322-1 35482211 PMC11073453

[76] Spiteri AG Terry RL Wishart CL Ashhurst TM Campbell IL Hofer MJ . High-parameter cytometry unmasks microglial cell spatio-temporal response kinetics in severe neuroinflammatory disease. J Neuroinflamm (2021) 18:166. doi: 10.1186/s12974-021-02214-y PMC8314570 34311763

[77] Perez-de-Puig I Miró-Mur F Ferrer-Ferrer M Gelpi E Pedragosa J Justicia C . Neutrophil recruitment to the brain in mouse and human ischemic stroke. Acta Neuropathol (2015) 129:239–57. doi: 10.1007/s00401-014-1381-0 25548073

[78] Garcia-Bonilla L Moore JM Racchumi G Zhou P Butler JM Iadecola C . Inducible nitric oxide synthase in neutrophils and endothelium contributes to ischemic brain injury in mice. J Immunol (2014) 193:2531–7. doi: 10.4049/jimmunol.1400918 PMC4147670 25038255

[79] Makinde HM Cuda CM Just TB Perlman HR Schwulst SJ . Nonclassical monocytes mediate secondary injury, neurocognitive outcome, and neutrophil infiltration after traumatic brain injury. J Immunol (2017) 199:3583–91. doi: 10.4049/jimmunol.1700896 PMC5679470 28993515

[80] Garcia-Bonilla L Racchumi G Murphy M Anrather J Iadecola C . Endothelial CD36 contributes to postischemic brain injury by promoting neutrophil activation via CSF3. J Neurosci (2015) 35:14783–93. doi: 10.1523/JNEUROSCI.2980-15.2015 PMC4635129 26538649

[81] Yao H-W Kuan C-Y . Early neutrophil infiltration is critical for inflammation-sensitized hypoxic-ischemic brain injury in newborns. J Cereb Blood Flow Metab (2020) 40:2188–200. doi: 10.1177/0271678X19891839 PMC7585929 31842667

[82] Pösel C Möller K Boltze J Wagner D-C Weise G . Isolation and flow cytometric analysis of immune cells from the ischemic mouse brain. J Visualized Experiments (2016) 108. doi: 10.3791/53658 PMC4828148 26967380

[83] García-Culebras A Durán-Laforet V Peña-Martínez C Moraga A Ballesteros I Cuartero MI . Role of TLR4 (Toll-like receptor 4) in N1/N2 neutrophil programming after stroke. Stroke (2019) 50:2922–32. doi: 10.1161/STROKEAHA.119.025085 31451099

[84] Roy-O’Reilly MA Ahnstedt H Spychala MS Munshi Y Aronowski J Sansing LH . Aging exacerbates neutrophil pathogenicity in ischemic stroke. Aging (2020) 12:436–61. doi: 10.18632/aging.102632 PMC6977697 31927534

[85] Mattei D Ivanov A van Oostrum M Pantelyushin S Richetto J Mueller F . Enzymatic dissociation induces transcriptional and proteotype bias in brain cell populations. Int J Mol Sci (2020) 21:7944. doi: 10.3390/ijms21217944 33114694 PMC7663484

[86] Herron S Delpech J-C Madore C Ikezu T . Using mechanical homogenization to isolate microglia from mouse brain tissue to preserve transcriptomic integrity. STAR Protoc (2022) 3:101670. doi: 10.1016/j.xpro.2022.101670 36107747 PMC9485281

[87] Calvo B Rubio F Fernández M Tranque P . Dissociation of neonatal and adult mice brain for simultaneous analysis of microglia, astrocytes and infiltrating lymphocytes by flow cytometry. IBRO Rep (2020) 8:36–47. doi: 10.1016/j.ibror.2019.12.004 32215337 PMC7090101

[88] Nahrendorf M Swirski FK . Abandoning M1/M2 for a network model of macrophage function. Circ Res (2016) 119:414–7. doi: 10.1161/CIRCRESAHA.116.309194 PMC4965179 27458196

[89] Herzenberg LA Tung J Moore WA Herzenberg LA Parks DR . Interpreting flow cytometry data: a guide for the perplexed. Nat Immunol (2006) 7:681–5. doi: 10.1038/ni0706-681 16785881

[90] Drucker AM Fleming P Chan A-W . Research techniques made simple: assessing risk of bias in systematic reviews. J Invest Dermatol (2016) 136:e109–14. doi: 10.1016/j.jid.2016.08.021 27772550

[91] Colonna M . TREMs in the immune system and beyond. Nat Rev Immunol (2003) 3:445–53. doi: 10.1038/nri1106 12776204

[92] Keating CE Houser MC MacPherson KP Herrick MK Coomes A Joers VL . Loss of progranulin leads to dysregulation of innate and adaptive immune cell populations, increased susceptibility to experimental colitis, and brain infiltration of peripheral immune cells. Alzheimer’s Dementia (2020) 16:1–2. doi: 10.1002/alz.042177

[93] Rhinn H Tatton N McCaughey S Kurnellas M Rosenthal A . Progranulin as a therapeutic target in neurodegenerative diseases. Trends Pharmacol Sci (2022) 43:641–52. doi: 10.1016/j.tips.2021.11.015 35039149

[94] Wallings RL Tansey MG . LRRK2 regulation of immune-pathways and inflammatory disease. Biochem Soc Trans (2019) 47:1581–95. doi: 10.1042/BST20180463 PMC6925522 31769472

[95] Golomb SM Guldner IH Zhao A Wang Q Palakurthi B Aleksandrovic EA . Multi-modal single-cell analysis reveals brain immune landscape plasticity during aging and gut microbiota dysbiosis. Cell Rep (2020) 33:108438. doi: 10.1016/j.celrep.2020.108438 33264626 PMC7737488

[96] Kovacs EJ Palmer JL Fortin CF Fülöp T Goldstein DR Linton P-J . Aging and innate immunity in the mouse: impact of intrinsic and extrinsic factors. Trends Immunol (2009) 30:319–24. doi: 10.1016/j.it.2009.03.012 PMC2898122 19541536

[97] Zhang X Pearsall VM Carver CM Atkinson EJ Clarkson BDS Grund EM . Rejuvenation of the aged brain immune cell landscape in mice through p16-positive senescent cell clearance. Nat Commun (2022) 13:5671. doi: 10.1038/s41467-022-33226-8 36167854 PMC9515187

[98] Erickson M Banks W . Age-associated changes in the immune system and blood–brain barrier functions. Int J Mol Sci (2019) 20:1632. doi: 10.3390/ijms20071632 30986918 PMC6479894

[99] Kalina T . Reproducibility of flow cytometry through standardization: opportunities and challenges. Cytometry Part A (2020) 97:137–47. doi: 10.1002/cyto.a.23901 31593368

[100] Hammond TR Dufort C Dissing-Olesen L Giera S Young A Wysoker A . Single-cell RNA sequencing of microglia throughout the mouse lifespan and in the injured brain reveals complex cell-state changes. Immunity (2019) 50:253–71.e6. doi: 10.1016/j.immuni.2018.11.004 30471926 PMC6655561

[101] Ritzel RM Lai Y-J Crapser JD Patel AR Schrecengost A Grenier JM . Aging alters the immunological response to ischemic stroke. Acta Neuropathol (2018) 136:89–110. doi: 10.1007/s00401-018-1859-2 29752550 PMC6015099

[102] Cumba Garcia LM Huseby Kelcher AM Malo CS Johnson AJ . Superior isolation of antigen-specific brain infiltrating T cells using manual homogenization technique. J Immunol Methods (2016) 439:23–8. doi: 10.1016/j.jim.2016.09.002 PMC5310589 27623324

[103] Srakočić S Josić P Trifunović S Gajović S Grčević D Glasnović A . Proposed practical protocol for flow cytometry analysis of microglia from the healthy adult mouse brain: Systematic review and isolation methods’ evaluation. Front Cell Neurosci (2022) 16:1017976. doi: 10.3389/fncel.2022.1017976 36339814 PMC9626753

[104] Molina Estevez FJ Mathews TD Biffi A Peviani M . Simultaneous flow cytometric characterization of multiple cell types retrieved from mouse brain/spinal cord through different homogenization methods. J Visualized Experiments (2019) 153. doi: 10.3791/60335 31814622

[105] Parks DR Moore WA Brinkman RR Chen Y Condello D El Khettabi F . Methodology for evaluating and comparing flow cytometers: A multisite study of 23 instruments. Cytometry Part A (2018) 93:1087–91. doi: 10.1002/cyto.a.23605 PMC7901711 30244531

[106] Lee JA Spidlen J Boyce K Cai J Crosbie N Dalphin M . MIFlowCyt: The minimum information about a flow cytometry experiment. Cytometry Part A (2008) 73A:926–30. doi: 10.1002/cyto.a.20623 PMC2773297 18752282

[107] Ruan C Elyaman W . A new understanding of TMEM119 as a marker of microglia. Front Cell Neurosci (2022) 16:902372. doi: 10.3389/fncel.2022.902372 35769325 PMC9234454

[108] Young KF Gardner R Sariana V Whitman SA Bartlett MJ Falk T . Can quantifying morphology and TMEM119 expression distinguish between microglia and infiltrating macrophages after ischemic stroke and reperfusion in male and female mice? J Neuroinflamm (2021) 18:58. doi: 10.1186/s12974-021-02105-2 PMC7901206 33618737

[109] Jurga AM Paleczna M Kuter KZ . Overview of general and discriminating markers of differential microglia phenotypes. Front Cell Neurosci (2020) 14:198. doi: 10.3389/fncel.2020.00198 32848611 PMC7424058

[110] Hopperton KE Mohammad D Trépanier MO Giuliano V Bazinet RP . Markers of microglia in post-mortem brain samples from patients with Alzheimer’s disease: a systematic review. Mol Psychiatry (2018) 23:177–98. doi: 10.1038/mp.2017.246 PMC5794890 29230021

[111] Fransen NL Hsiao C-C van der Poel M Engelenburg HJ Verdaasdonk K Vincenten MCJ . Tissue-resident memory T cells invade the brain parenchyma in multiple sclerosis white matter lesions. Brain (2020) 143:1714–30. doi: 10.1093/brain/awaa117 32400866

[112] Steinbach K Vincenti I Kreutzfeldt M Page N Muschaweckh A Wagner I . Brain-resident memory T cells represent an autonomous cytotoxic barrier to viral infection. J Exp Med (2016) 213:1571–87. doi: 10.1084/jem.20151916 PMC4986533 27377586

[113] Prasad S Lokensgard JR . Brain-resident T cells following viral infection. Viral Immunol (2019) 32:48–54. doi: 10.1089/vim.2018.0084 30230418 PMC6350058

[114] Sedgwick AJ Ghazanfari N Constantinescu P Mantamadiotis T Barrow AD . The role of NK cells and innate lymphoid cells in brain cancer. Front Immunol (2020) 11:1549. doi: 10.3389/fimmu.2020.01549 32903717 PMC7438769

[115] Cuapio A Ljunggren H-G . Activated natural killer cells hit neurogenesis in the aging brain. Neurosci Bull (2021) 37:1072–4. doi: 10.1007/s12264-021-00654-3 PMC8006623 33779894

[116] Earls RH Lee JK . The role of natural killer cells in Parkinson’s disease. Exp Mol Med (2020) 52:1517–25. doi: 10.1038/S12276-020-00505-7 PMC8080760 32973221

[117] McKenna E Mhaonaigh AU Wubben R Dwivedi A Hurley T Kelly LA . Neutrophils: need for standardized nomenclature. Front Immunol (2021) 12:602963. doi: 10.3389/fimmu.2021.602963 33936029 PMC8081893

[118] Fine N Barzilay O Sun C Wellappuli N Tanwir F Chadwick JW . Primed PMNs in healthy mouse and human circulation are first responders during acute inflammation. Blood Adv (2019) 3:1622–37. doi: 10.1182/bloodadvances.2018030585 PMC6538871 31138591

[119] McGill CJ Lu RJ Benayoun BA . Protocol for analysis of mouse neutrophil NETosis by flow cytometry. STAR Protoc (2021) 2:100948. doi: 10.1016/j.xpro.2021.100948 34820637 PMC8599168

[120] Manda-Handzlik A Ostafin M Bystrzycka W Sieczkowska S Moskalik A Demkow U . Flow cytometric quantification of neutrophil extracellular traps: Limitations of the methodological approach. Am J Hematol (2016) 91:E9–E10. doi: 10.1002/ajh.24257 26616047

[121] Sakata-Kaneko S Wakatsuki Y Matsunaga Y Usui T Kita T . Altered Th1/Th2 commitment in human CD4+ T cells with ageing. Clin Exp Immunol (2001) 120:267–73. doi: 10.1046/j.1365-2249.2000.01224.x PMC1905644 10792375

[122] D’Agostino PM Gottfried-Blackmore A Anandasabapathy N Bulloch K . Brain dendritic cells: biology and pathology. Acta Neuropathol (2012) 124:599–614. doi: 10.1007/s00401-012-1018-0 22825593 PMC3700359

[123] Constant O Maarifi G Blanchet FP Van de Perre P Simonin Y Salinas S . Role of dendritic cells in viral brain infections. Front Immunol (2022) 13:862053. doi: 10.3389/fimmu.2022.862053 35529884 PMC9072653

[124] Ludewig P Gallizioli M Urra X Behr S Brait VH Gelderblom M . Dendritic cells in brain diseases. Biochim Biophys Acta (BBA) - Mol Basis Dis (2016) 1862:352–67. doi: 10.1016/j.bbadis.2015.11.003 26569432

[125] Clark J McFarlane C Cleo G Ishikawa Ramos C Marshall S . The impact of systematic review automation tools on methodological quality and time taken to complete systematic review tasks: case study. JMIR Med Educ (2021) 7:e24418. doi: 10.2196/24418 34057072 PMC8204237

[126] Marshall IJ Wallace BC . Toward systematic review automation: a practical guide to using machine learning tools in research synthesis. Syst Rev (2019) 8:163. doi: 10.1186/s13643-019-1074-9 31296265 PMC6621996

